# Brugada Phenocopy Caused by Intracranial Hemorrhage

**DOI:** 10.7759/cureus.35687

**Published:** 2023-03-02

**Authors:** Tiago Branco, Ana Barbosa, Nelson Cunha, João Gouveia, João M Lopes

**Affiliations:** 1 Serviço de Medicina 2, Hospital de Santa Maria, Centro Hospitalar Universitário Lisboa Norte, Lisboa, PRT; 2 Departament de Cardiologia, Hospital de Santa Maria, Centro Hospitalar Universitário Lisboa Norte, Centro Académico de Medicina de Lisboa, Centro Cardiovascular da Universidade de Lisboa, Faculdade de Medicina de Lisboa, Universidade de Lisboa, Lisboa, PRT; 3 Serviço de Medicina Intensiva, Hospital de Santa Maria, Centro Hospitalar Universitário Lisboa Norte, Lisboa, PRT

**Keywords:** traumatic subarachnoid hemorrhage, acute subdural hematoma, elevated intracranial pressure, brugada phenocopy, brugada ecg pattern

## Abstract

Brugada syndrome (BrS) is a congenital channelopathy associated with an increased risk of malignant ventricular arrhythmias and sudden cardiac death in individuals without any structural cardiopathy. Brugada phenocopies (BrPs) are clinical entities that present electrocardiographic patterns similar to those of BrS that are elicited only under transitory pathophysiological conditions, with normalization of the ECG pattern after the resolution of those conditions. We present a rare case of BrP due to intracranial hemorrhage. We also present and discuss the diagnostic criteria for BrPs and their application to this case.

## Introduction

Brugada syndrome (BrS) is a congenital channelopathy associated with an increased risk of malignant ventricular arrhythmias and sudden cardiac death in individuals without any structural cardiopathy [1]. It was first described in 1992 by Pedro and Josep Brugada in a report concerning eight individuals resuscitated from sudden cardiac death caused by documented ventricular fibrillation [2]. The pathophysiology of BrS is associated with the loss of function of cardiomyocyte sodium channels and has a genetic background, with mutations in SCN5A (Human Gene Mutation Database) reported in most pathogenic variants reported so far [1].

BrS is characterized by two types of specific electrocardiographic (ECG) patterns. Type 1 morphology consists of a coved ST-elevation ($\geq$2 mm) in one or more of the right precordial leads, V1 and/or V2, positioned in the second, third, or fourth intercostal spaces, followed by an r’-wave and a concave or straight ST segment. The descending ST segment crosses the isoelectric line and is followed by a negative and symmetric T-wave [1]. Type 2 (or saddle-back type) morphology is characterized by an ST-segment elevation ($\geq$0.5 mm) in one or more of the right precordial leads (V1 to V3), followed by a convex ST. The ST segment is followed by a positive T-wave in V2 (with variable morphology in V1) [1]. Additional criteria have been proposed to facilitate the differentiation between BrS and other Brugada-like patterns [3,4].

Diagnosing BrS requires a clinical history in the presence of a type 1 ECG pattern [5]. This type 1 pattern can either occur spontaneously or be induced after a provocative drug test with intravenous administration of sodium-channel blockers [1].

The presence of all other known causes of ST-segment elevation in right precordial leads (known as phenocopies) should be excluded before making the diagnosis of BrS. Brugada phenocopies (BrPs) are clinical entities that present type 1 or type 2 electrocardiographic patterns similar to BrS that are elicited only under transitory pathophysiological conditions, with normalization of the ECG patterns after resolution of those conditions [6].

Several categories of etiologies have been proposed for BrPs. The conditions commonly associated with the occurrence of BrPs are metabolic conditions, mechanical compression of the heart, ischemia and pulmonary embolism, myocardial and pericardial disease, ECG modulation, and a residual miscellaneous group of conditions that could not be classified into any of the previous groups [5,7]. These include Ebstein anomaly, intracranial hemorrhage, and status post-biphasic synchronized electrical cardioversion for atrial fibrillation [5].

Besides all the aforementioned etiological categories that are linked to the occurrence of BrPs, two subgroups are not classified as BrPs: the fever-induced Brugada pattern and the Brugada pattern induced by drugs with sodium channel blockers [7,8]. The BrP pattern caused by these two etiology subgroups cannot be diagnosed as BrP, even if all other diagnostic criteria are met, given that the underlying pathophysiological mechanisms and prognosis might differ from those of BrP [5].

## Case presentation

**Figure 1:**
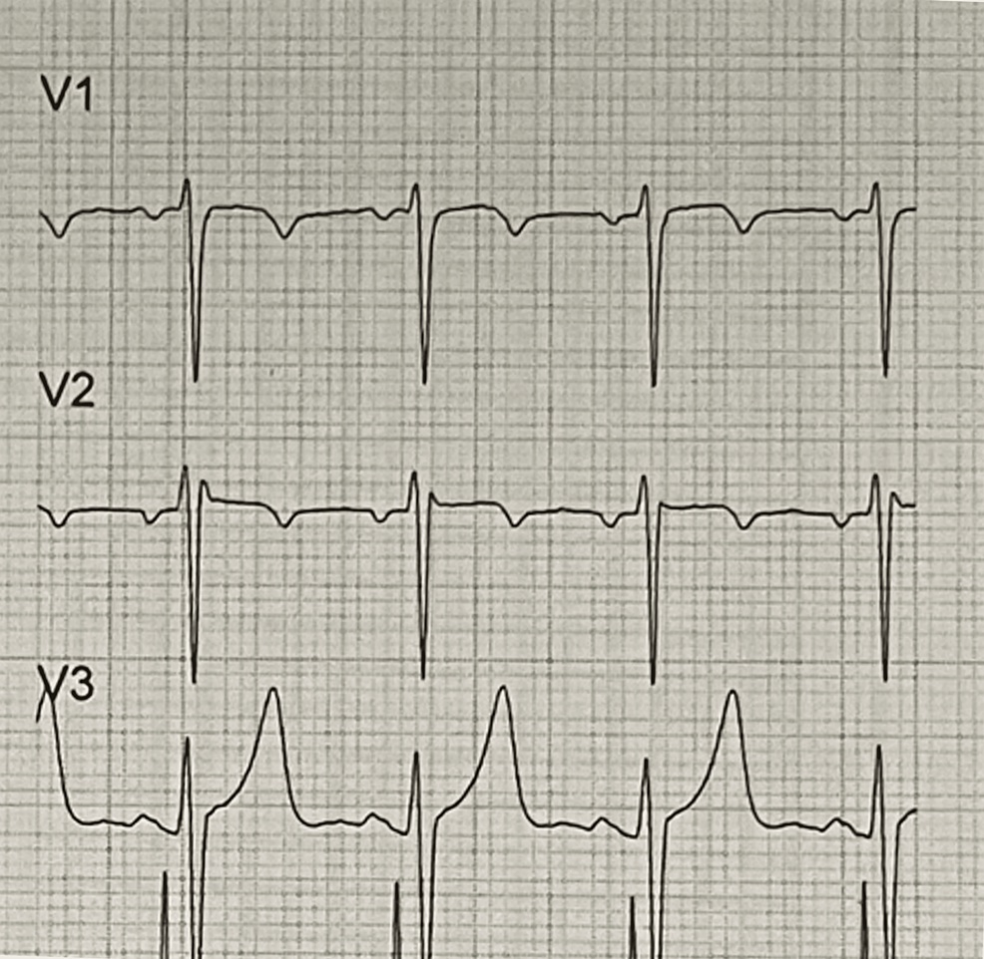
V1-V3 precordial leads upon admission.

A 21-year-old man with no significant clinical history was admitted to the emergency room (ER) after a high-kinetic collision with a car while riding a horse. The accident resulted in polytrauma with serious head trauma, with a reported onsite Glasgow Coma Scale score of 6. He was sedated, ventilated, and transported to the ER. A head CT performed after admission revealed multiple fractures in the base of the skull and fractures of the left half frontal bone associated with a frontal left extra-axial hematic collection molding the adjacent convexity parenchyma. A falcotentorial subdural hematoma (SDH) with no mass effect and a Sylvian fissure subarachnoid hemorrhage (SAH) were also observed. In addition, he presented with bitemporal and left occipital contusion focuses, a cerebrospinal fluid nasal fistula, and multiple other body fractures. The ECG performed at admission was near normal (Figure 1).

An external ventricular drain was placed for intracranial pressure (ICP) management, and the patient was admitted to the neurocritical care ward for neuroprotection measures with an indication to keep ICP to a maximum of 15 mm Hg. His condition worsened in the following days, marked by frequent episodes of elevated ICP (max. 22 mm Hg) requiring sedation with thiopental and remifentanil and occasional hypertonic therapeutics.

**Figure 2:**
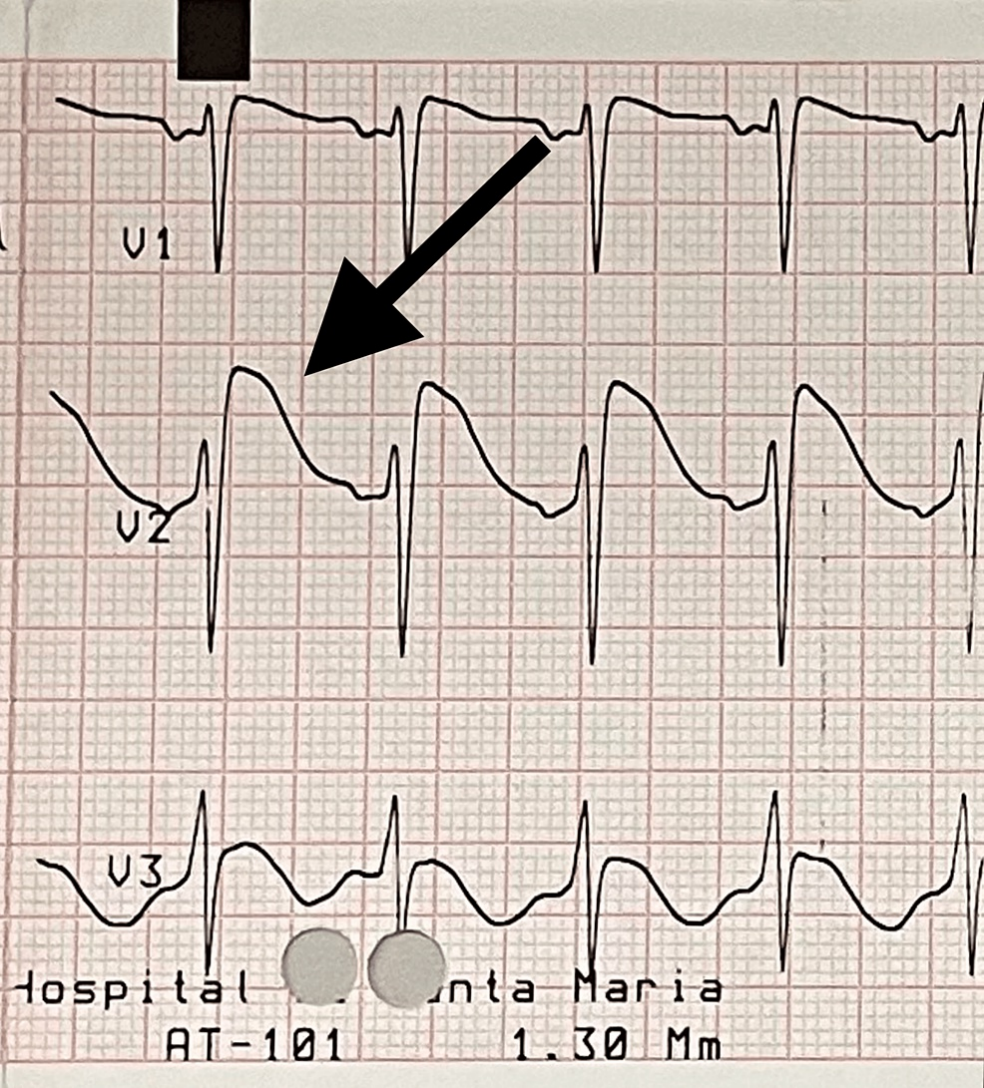
V1-V3 precordial leads at day 8 after admission.

On the eighth day after admission, a type 1 Brugada pattern was documented (Figure 2).

At this point, the patient was afebrile, and, apart from frequent ICP elevations, no other conditions associated with transitory BrPs were present. A bedside echocardiogram was performed without abnormal findings.

**Figure 3:**
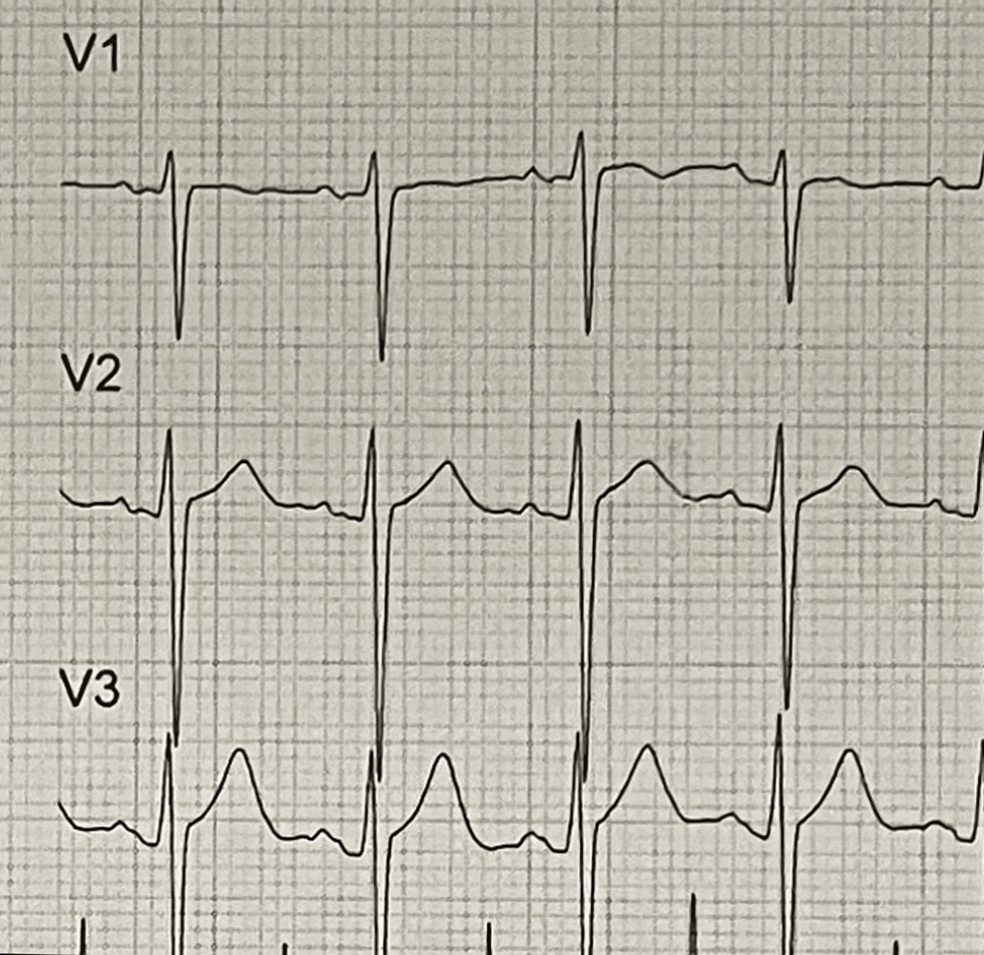
V1-V3 precordial leads at day 12 after admission.

On the 12th day after admission, when the ICP elevation episodes had subsided, a resolution to the initial normal ECG without a Brugada pattern was documented (Figure 3), with no further changes after that period. Between days 8 and 12, sedation was weaned based on the progress of the patient’s condition; however, no changes were made to the drugs prescribed.

Subsequent imaging exams on day 12 showed a reduction in the sizes of both the SDH and SAH and revealed no other complications. The patient’s condition eventually became stable enough for him to be transferred to the neurosurgery ward. He was discharged 39 days after admission and started a long rehabilitation process. A few months later, the patient was summoned to perform a provocative test with ajmaline, which resulted in a negative.

## Discussion

A definitive diagnosis of BrP requires the following criteria to be met: (1) type 1 or type 2 Brugada morphologic criteria met on ECG; (2) presence of an identifiable and reversible underlying condition; (3) resolution of the ECG pattern on the resolution of the underlying condition; (4) low pretest probability for Brugada syndrome; (5) a negative provocative test with a sodium channel blocker; and (6) a negative genetic test [5]. The first four criteria are mandatory to be met for a diagnosis of BrPs. The provocative test is mandatory unless the patient underwent a surgical right ventricular outflow tract manipulation within the last 96 hours. Genetic testing is not mandatory given the SCN5A mutation has been identified in only 30% of probands with true Brugada syndrome [1].

Several circumstances may preclude satisfaction of all criteria, such as patient death, loss of follow-up, or unwillingness to undergo further testing. Therefore, BrP cases are further classified as A, B, or C [6]. Cases that satisfy all criteria, including a provocative challenge with a sodium channel blocker, are categorized as class A. Cases wherein BrP is highly suspected but not all diagnostic criteria are met are categorized as class B. Cases wherein a provocative test with sodium channel blockers is not required because the cause of the ECG Brugada pattern is easily reproducible or identifiable, such as a recent surgery, are classified as class C [6].

Our patient presented a transitory type 1 ECG pattern while experiencing acute encephalopathy with high ICP peaks in the context of head trauma with a documented falcotentorial subdural hematoma and a Sylvian fissure subarachnoid hemorrhage.

Several cardiac complications of neurologic disease causing transitory Brugada patterns are known; however, not all meet the criteria for a BrP diagnosis. Acute encephalopathy and disorders such as intracerebral or subarachnoid hemorrhage have been reported to induce ECG ST-segment changes, including Brugada-like patterns [9]. However, in many of the reported cases, other concomitant conditions were also present (such as patients being febrile or receiving propofol infusions), leading to the exclusion of the diagnosis of BrP [10].

However, at least one reported case of true BrP caused by intracranial hemorrhage exists [10], which we believe was the case with our patient. During the period of documented type 1 pattern ECG changes, the patient was apyretic, and no other conditions or agents known to modulate BrS or transitory BrPs were present [8]. We admit that the persisting ICP elevation episodes may have increased the effect of the documented SDH and SAH. Once the lesions regressed, the ECG pattern normalized.

Our patient also presented with a low pretest probability for BrS. There was no personal history of syncope or aborted sudden cardiac death. Family history also did not include any diagnosis of Brugada patterns, episodes of life-threatening arrhythmias, implantation of cardioverter-defibrillators, or unexpected deaths. The provocative test with a sodium channel blocker (ajmaline) was negative. For the aforementioned reasons, we believe this case qualifies as a class A BrP.

## Conclusions

True BrPs related to intracranial hemorrhage are very rare. We present the case of a young man who experienced violent head trauma resulting in a falcotentorial subdural hematoma and a Sylvian fissure subarachnoid hemorrhage associated with elevated intracranial pressure. We observed a type 1 ECG pattern that was not present at the admission, which resolved after the size reduction of the hematic collections. During this period, no other conditions or agents known to modulate BrP were present, and the patient presented a negative provocative test. Therefore, we believe this case qualifies as a rare case of class A BrP due to the presence of intracranial hemorrhage.
